# Optimal waist circumference cut-off points and ability of different metabolic syndrome criteria for predicting diabetes in Japanese men and women: Japan Epidemiology Collaboration on Occupational Health Study

**DOI:** 10.1186/s12889-016-2856-9

**Published:** 2016-03-03

**Authors:** Huanhuan Hu, Kayo Kurotani, Naoko Sasaki, Taizo Murakami, Chii Shimizu, Makiko Shimizu, Tohru Nakagawa, Toru Honda, Shuichiro Yamamoto, Hiroko Okazaki, Satsue Nagahama, Akihiko Uehara, Makoto Yamamoto, Kentaro Tomita, Teppei Imai, Akiko Nishihara, Takeshi Kochi, Masafumi Eguchi, Toshiaki Miyamoto, Ai Hori, Keisuke Kuwahara, Shamima Akter, Ikuko Kashino, Isamu Kabe, Weiping Liu, Tetsuya Mizoue, Naoki Kunugita, Seitaro Dohi, the Japan Epidemiology Collaboration on Occupational Health Study Group

**Affiliations:** Department of Epidemiology and Prevention, Center for Clinical Sciences, National Center for Global Health and Medicine, Toyama 1-21-1, Shinjuku-ku, Tokyo 162-8655 Japan; Mitsubishi Fuso Truck and Bus Corporation, Kanagawa, Japan; Mizue Medical Clinic, Keihin Occupational Health Center, Kanagawa, Japan; Hitachi, Ltd., Ibaraki, Japan; Mitsui Chemicals, Inc., Tokyo, Japan; All Japan Labour Welfare Foundation, Tokyo, Japan; YAMAHA CORPORATION, Shizuoka, Japan; Mitsubishi Plastics, Inc., Tokyo, Japan; Azbil Corporation, Tokyo, Japan; Furukawa Electric Co., Ltd., Tokyo, Japan; Nippon Steel & Sumitomo Metal Corporation Kimitsu Works, Chiba, Japan; Tokyo Gas Co., Ltd., Tokyo, Japan; Teikyo University Graduate School of Public Health, Tokyo, Japan; Xian Janssen Pharmaceutical Ltd., Shanghai, China; National Institute of Public Health, Saitama, Japan

**Keywords:** Waist circumference, Metabolic syndrome, Diabetes mellitus

## Abstract

**Background:**

We sought to establish the optimal waist circumference (WC) cut-off point for predicting diabetes mellitus (DM) and to compare the predictive ability of the metabolic syndrome (MetS) criteria of the Joint Interim Statement (JIS) and the Japanese Committee of the Criteria for MetS (JCCMS) for DM in Japanese.

**Methods:**

Participants of the Japan Epidemiology Collaboration on Occupational Health Study, who were aged 20–69 years and free of DM at baseline (*n* = 54,980), were followed-up for a maximum of 6 years. Time-dependent receiver operating characteristic analysis was used to determine the optimal cut-off points of WC for predicting DM. Time-dependent sensitivity, specificity, and positive and negative predictive values for the prediction of DM were compared between the JIS and JCCMS MetS criteria.

**Results:**

During 234,926 person-years of follow-up, 3180 individuals developed DM. Receiver operating characteristic analysis suggested that the most suitable cut-off point of WC for predicting incident DM was 85 cm for men and 80 cm for women. MetS was associated with 3–4 times increased hazard for developing DM in men and 7–9 times in women. Of the MetS criteria tested, the JIS criteria using our proposed WC cut-off points (85 cm for men and 80 cm for women) had the highest sensitivity (54.5 % for men and 43.5 % for women) for predicting DM. The sensitivity and specificity of the JCCMS MetS criteria were ~37.7 and 98.9 %, respectively.

**Conclusion:**

Data from the present large cohort of workers suggest that WC cut-offs of 85 cm for men and 80 cm for women may be appropriate for predicting DM for Japanese. The JIS criteria can detect more people who later develop DM than does the JCCMS criteria.

**Electronic supplementary material:**

The online version of this article (doi:10.1186/s12889-016-2856-9) contains supplementary material, which is available to authorized users.

## Background

Metabolic syndrome (MetS) is defined as a clustering of metabolic abnormalities [1], and has been shown to be associated with increased risks of developing type 2 diabetes mellitus (DM) and cardiovascular disease [2–4]. However, controversy exist over the diagnostic criteria and utility of MetS [5–7]. Of several MetS criteria proposed [5, 8], the National Cholesterol Education Program Adult Treatment Panel III (NCEP-ATP III) [1] and the International Diabetes Federation (IDF) [9] have been most widely used. In 2009, a Joint Interim Statement (JIS) criteria was published as a combined effort by a number of international scientific bodies and authorities [8]. Besides these international definitions, a Japanese-specific MetS criteria has been proposed by the Japanese Committee of the Criteria for MetS (JCCMS) [10].

These criteria differ in several aspects, including the cut-off points of waist circumference (WC), handling of the WC component (prerequisite or optional for the diagnosis of MetS), and the criteria of hyperglycemia and dyslipidemia. These differences have led to confusion regarding the choice of the criteria to diagnose MetS. One major concern for Japanese individuals is the WC cut-off point. The JCCMS adopts cut-offs of WC ≥85 cm for men and ≥90 cm for women [10]. In Japan, cross-sectional studies have suggested cut-off points of 85–90 cm for men and 77–83 cm for women for predicting the presence of multiple other MetS components [11–13], and a recent prospective study showed that the optimal cut-off point of WC for predicting cardiovascular disease (CVD) was 90 cm in men and 80 cm in women [14]. Several prospective studies have examined the ethnic specific WC cut-off values in relation to DM risk among Koreans, Mexicans, and Americans [15–17]; however, no such study has yet been performed in the Japanese population.

Here, we examined the optimal WC cut-off points for predicting the development of DM and compared the predictive ability of two MetS criteria (JIS [8] and JCCMS [10]) in a large-scale working population in Japan.

## Methods

### Survey description

The Japan Epidemiology Collaboration on Occupational Health (J-ECOH) Study is an ongoing multicenter epidemiologic study among workers from several companies in Japan. A total of 12 companies covering various industries (electric machinery and apparatus manufacturing; steel, chemical, gas, and non-ferrous metal manufacturing; automobile and instrument manufacturing; plastic product manufacturing; and health care) participated in the J-ECOH study. As of May 2014, eleven participating companies provided health check-up data obtained between January 2008 and December 2013 or between April 2008 and March 2014. In this study, the data from the earliest examination (mostly carried out in 2008) were regarded as the baseline data; however, if the 2008 dataset contained a large number of missing data, the data from the 2009 or 2010 (one company each) examination were used as the baseline. The outcomes of the present prospective analysis were determined using data from the health check-ups after the baseline through 2014.

The J-ECOH Study was announced in each company using posters. In Japan, workers are obliged to undergo health examination at least once a year under the Industrial Safety and Health Act; nearly all workers attend their health examination in each year. Participants did not provide their oral or written informed consent to take part in the study but were given an opportunity to refuse the use of their data for research, according to the Japanese Ethical Guidelines for Epidemiological Research [18]. The details of the J-ECOH Study have been described elsewhere [19, 20]. The study protocol was approved by the Ethics Committee of the National Center for Global Health and Medicine, Japan.

### Study participants

**Fig. 1:**
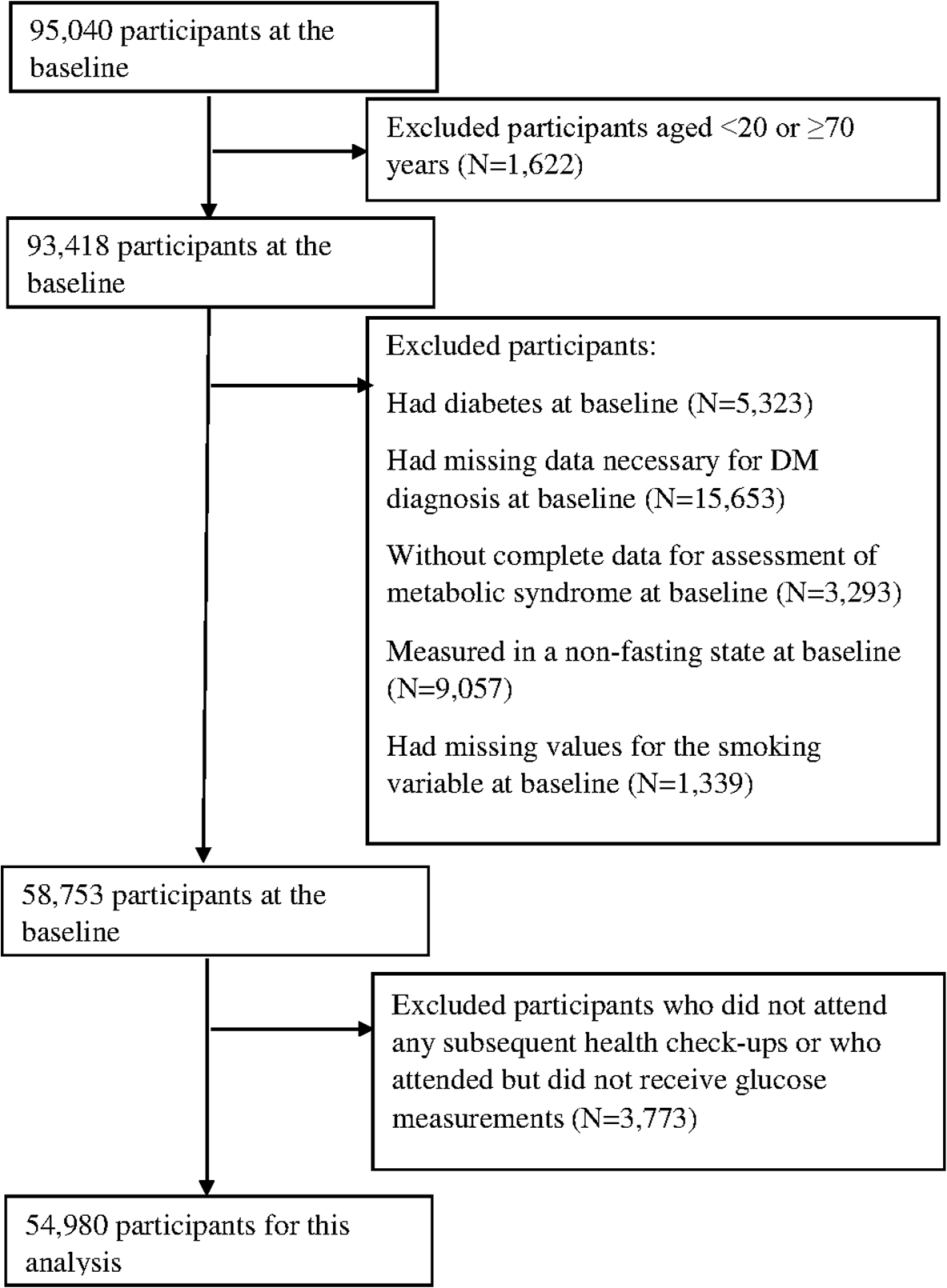
Flow chart of participant selection

As shown in Fig. 1, of 95,040 participants who attended the baseline health check-up, we excluded those who were aged <20 or ≥70 years, who had DM or missing data necessary for the diagnosis of DM or MetS, and who attended the health check-up in a non-fasting state or lacked information on smoking. Of the remaining 58,753 participants, we further excluded those who did not attend any subsequent health check-up or who attended but did not receive glucose measurements, leaving 54,980 participants (46,981 men and 7999 women) for analysis.

### Measurements

The body height, body weight, WC, and blood pressure were measured according to a standard protocol of each company. Body mass index (BMI) was calculated as the weight in kilograms divided by the squared height in meters. WC was measured at the umbilical level using a measuring tape, with the subjects in the standing position [21]. Blood pressure was measured in a sitting position. Hypertension was defined as systolic blood pressure ≥140 mmHg, diastolic blood pressure ≥90 mmHg, or as receiving medical treatment for hypertension [22]. Smoking status was ascertained via a self-administered questionnaire.

The plasma glucose was measured by the enzymatic or glucose oxidase peroxidative electrode method. Glycated hemoglobin (HbA1c) was measured by using latex agglutination immunoassay, high-performance liquid chromatography, or the enzymatic method. Total cholesterol (TCH), triglyceride (TG), low-density lipoprotein-cholesterol (LDL-C), and high-density lipoprotein-cholesterol (HDL-C) level were measured by the enzymatic method. All laboratories involved in the health checkup in the participating companies have received satisfactory scores (rank A or a score >95 out of 100) from external quality control agencies.

### Criteria of MetS

We defined the presence of MetS using the following two criteria (Table 1): JIS [8] and JCCMS [10].

**Table 1:** Diagnostic criteria for metabolic syndrome

Risk factor	JIS	JCCMS
WC	M ≥ 90 Cm W ≥ 80 cm	M ≥ 85 cm W ≥ 90 cm
BP	SBP ≥ 130 and/or DBP ≥ 85 mmHg or on treatment for HPT	SBP ≥ 130 and/or DBP ≥ 85 mmHg or on treatment for HPT
FPG	≥100 mg/dl or diagnosed DM	≥110 mg/dl or diagnosed DM
TG	≥150 mg/dl or Treatment for TG	TG ≥150 mg/dl or treatment for TG or HDL < 40 mg/dl
HDL-C	M < 40 mg/dl, W < 50 mg/dl or treatment for HDL	
Criteria	At least 3	WC + 2 or more

*WC* waist circumference, *FPG* fasting plasma glucose, *TG* triglyceride, *HDL-C* high-density lipoprotein cholesterol, *SBP* systolic blood pressure, *DBP* diastolic blood pressure, *M* men, *W* women, *DM* diabetes mellitus, *HPT* hypertension, *Modified NCEP-ATP III* National Cholesterol Education Program Adult Treatment Panel III (2005), *JIS* the Joint Interim Statement on metabolic syndrome definition (2009), *JCCMS* Japanese Committee of the Criteria for Metabolic Syndrome (2005)

### Outcome

DM was identified using data from the annual health check-ups for a maximum of 6 years after the baseline examination. DM was defined as HbA1c ≥6.5 %, fasting plasma glucose ≥126 mg/dl, random plasma glucose ≥200 mg/dl, or currently under medical treatment for DM, according to the American Diabetes Association criteria for the diagnosis of DM [23]. Individuals without DM at baseline who met any of the above conditions in the subsequent check-ups were considered to have an incident case of type 2 DM.

### Statistical analyses

All analyses were performed separately by sex. Descriptive statistics were generated using the sample size, percentage, and mean. Chi-square tests for categorical variables or t-tests for continuous variables were used to examine differences in baseline characteristics between subjects with incident DM and those who did not develop it. Age-standardized incidence of DM and mean WC for each company were calculated by direct standardization to the total population of the present study. Time-dependent receiver operating characteristic (ROC) curves for WC for predicting the development of DM in the next 5 years were depicted with the Kaplan-Meier method [24]. The optimal WC cut-offs on the ROC curve were determined by applying the Youden’s index [25] and the closest top-left point approaches [26].

Person-time was calculated from the date of the baseline examination to the date of the first diagnosis of DM in a subsequent examination or to the date of the last examination, whichever occurred first. Cox proportional hazards regression models were used to estimate the hazard ratios (HRs) for the development of DM associated with MetS in men and women, respectively. Covariates included age (years) and tobacco smoking (current smoker, non-current smoker). Company was treated as a cluster variable to account for intraclass correlations. The time-on-study was used as the primary time scale. We verified that the proportional hazards assumption was not violated for our main exposure and other covariates by including interaction terms with time, and we used the Wald chi-square procedure to test whether all coefficients equaled 0. To compare the predictive ability of the JIS and the JCCMS MetS criteria, the sensitivities, specificities, and positive and negative predictive values at 5-year follow-up were determined [27].

The JCCMS criteria requires WC as a prerequisite for the diagnosis of MetS [10]. Hence, additional analyses were performed to determine if the sensitivity and specificity of JCCMS could be improved when having WC as an “optional” rather than “essential” criterion (modified JCCMS criteria).

The time-dependent sensitivities, specificities, and positive and negative predictive values for different criteria of MetS were performed using R version 3.2.2 (R Foundation for Statistical Computing, Vienna, Austria). All other statistical analyses were performed using SAS version 9.3 (SAS Institute, Cary, NC, USA). A two-sided *P* <0.05 was considered statistically significant.

## Results

During 234,926 person-years of follow-up (a median of 4.9 years), 3180 individuals (2963 men and 217 women) developed DM. The incidence rate of progression to DM was 14.7 per 1000 person-years of observation for men, and 6.5 per 1000 person-years for women. The age-standardized incidence of DM across companies ranged between 9 and 15 per 1000 person-years for men, and between 6 and 7 per 1000 person-years for women.

The baseline characteristics of the subjects according to the presence or absence of incident DM are shown in Table 2. For both sexes, the mean age, BMI, WC, systolic blood pressure, diastolic blood pressure, fasting plasma glucose, TCH, TG, and LDL-C in subjects who developed DM were greater than in those who did not, while the HDL-C was lower. Moreover, the prevalence rates of hypertension and smoking were higher in subjects with incident DM than in those who did not develop it for both men and women. The coefficients between BMI and WC were 0.88 for men and 0.84 for women. The age-standardized mean WC across companies ranged between 81.0 and 84.0 cm for men, and between 74.0 and 77.0 cm for women. The characteristics of the study participants by MetS and sex are shown in Additional file 1.

**Table 2:** Baseline characteristics of subjects

	Men		Women	
	No DM	DM	No DM	DM
N	44018	2963	7782	217
Age (years)	45.4 ± 8.9	49.2 ± 7.6*	43.9 ± 8.9	48.4 ± 7.8*
BMI (kg/m^2^)	23.4 ± 3.0	25.3 ± 3.7*	21.5 ± 3.3	25.3 ± 5.4*
WC (cm)	82.9 ± 8.2	88.0 ± 9.3*	75.4 ± 8.9	84.3 ± 11.8*
FPG (mg/dl)	96.3 ± 8.3	109.2 ± 9.7*	90.8 ± 7.8	104.1 ± 10.2*
TCH (mg/dl)^a^	200.9 ± 32.6	208.2 ± 33.3*	200.8 ± 33.5	215.7 ± 34.2*
TG (mg/dl)	125.5 ± 90.1	159.7 ± 124.4*	74.9 ± 41.6	121.7 ± 112.7*
LDL-C (mg/dl)	120.2 ± 29.5	126.4 ± 30.5*	113.7 ± 29.9	128.7 ± 32.7*
HDL-C (mg/dl)	57.1 ± 14.4	53.5 ± 13.9*	69.6 ± 15.5	63.4 ± 17.1*
SBP (mmHg)	121.5 ± 14.8	128.1 ± 16.2*	115.3 ± 16.0	127.4 ± 18.3*
DBP (mmHg)	77.1 ± 10.4	81.5 ± 10.7*	71.6 ± 10.6	78.6 ± 11.4*
Hypertension (%)	19.7	36.8*	10.1	31.8*
Smoking (%)	40.5	45.8*	10.7	15.2*

Data was expressed as mean ± SD or as percentages

*DM* diabetes mellitus, *BMI* body mass index, *WC* waist circumference, *FPG* fasting plasma glucose, *TCH* Total cholesterol, *TG* triglyceride, *LDL-C* low-density lipoprotein cholesterol, *HDL-C* high-density lipoprotein cholesterol, *SBP* systolic blood pressure, *DBP* diastolic blood pressure

*Difference between groups is statistically significant (*P* < 0.05)

^a^Data were available for 46,152 subjects

### Optimal WC cut-off points

**Fig. 2:**
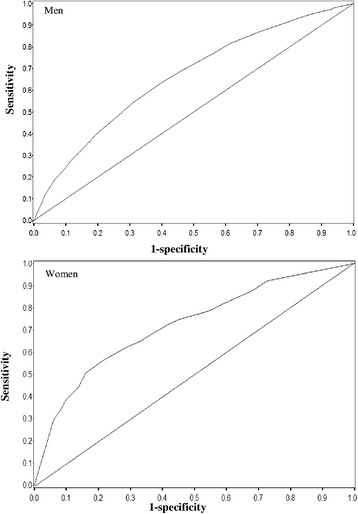
ROC curves for the prediction of diabetes in the next 5 years using baseline WC. ROC curves showing the ability of baseline waist circumference (WC) to predict the development of diabetes in the next 5 years

Figure 2 shows ROC curves for the prediction of the development of DM within the next 5 years using baseline WC. The AUCs were 0.67 (95 % confidence interval [CI], 0.61–0.72) for men, and 0.70 (0.62–0.78) for women. The optimal cut-off points of WC according to the ROC curve for predicting incident DM for men and women are shown in Table 3. In men, a cut-off point of 85.0 cm (sensitivity 64.0 %, specificity 59.6 %) yielded both the maximal Youden index and minimum distance from the top left corner of the ROC curve. In women, a cut-off point of 83.0 cm (sensitivity 55.5 %, specificity 79.4 %) yielded the maximal Youden index, and 80.0 cm (62.8 %, 70.7 %) yielded the minimum distance from the top left corner of the ROC curve. Taken together with earlier studies in Japan [11–13], we thus propose WC cut-off points of 85 cm for men and 80 cm for women.

**Table 3:** Performance of different waist thresholds to predict the development of diabetes in the next 5 years

Waist thresholds	Sensitivity	Specificity	Youden index	Distance^a^
Men				
97.0 cm	16.2 %	94.6 %	0.108	0.840
95.0 cm	18.7 %	93.5 %	0.122	0.816
90.0 cm	35.0 %	83.5 %	0.185	0.671
88.0 cm	49.1 %	73.0 %	0.221	0.576
85.0 cm	64.0 %	59.6 %	0.235	0.541
83.0 cm	68.6 %	54.2 %	0.228	0.555
80.0 cm	81.4 %	38.8 %	0.202	0.640
Women				
90.0 cm	29.3 %	94.0 %	0.234	0.709
87.0 cm	38.8 %	90.0 %	0.287	0.621
84.0 cm	50.6 %	84.0 %	0.346	0.519
83.0 cm	55.5 %	79.4 %	0.349	0.490
80.0 cm	62.8 %	70.7 %	0.334	0.474
78.0 cm	64.9 %	67.0 %	0.319	0.482
75.0 cm	75.0 %	54.6 %	0.296	0.518

^a^Distance, the distance from a point on the curve to the point (0, 1)

### Association between MetS and DM

The prevalence of MetS ranged from 12.0 to 22.1 % in men and 1.3 to 8.0 % in women according to the different criteria of MetS (Table 4). The incidence rates of DM were higher in subjects with MetS than in those without for both sexes (*P* < 0.05). In men, the adjusted HRs (95 % CI) for the development of DM according to the different criteria of MetS were as follows: JIS (Asian WC cut-off value) 3.72 (3.46–4.00), JIS (proposed WC cut-off value) 3.84 (3.69–4.00), and JCCMS (same for Japanese central obesity criteria and proposed WC cut-off point) 4.16 (3.89–4.45). In women, the adjusted HRs were as follows: JIS (same for Asian WC cut-off value and proposed WC cut-off value) 7.08 (6.19–8.09), JCCMS (Japanese central obesity criteria) 9.63 (8.17–11.36), and JCCMS (proposed WC cut-off point) 7.40 (5.68–9.64). Kaplan-Meier estimates of DM-free survival by MetS and sex are provided in Additional file 2.

**Table 4:** Risk for the development of diabetes associated with metabolic syndrome

		Incidence of diabetes per 1000 person-years		
Metabolic syndrome	Prevalence at baseline (%)	Without	With	Adjusted HR (95 % CI) for incident DM^a^
JIS (Asian cut-off points for WC)				
Men	16.5	9.7	42.0	3.72 (3.46–4.00)
Women	8.0	4.1	37.1	7.08 (6.19–8.09)
JIS (Proposed cut-off points for WC)				
Men	22.1	8.5	38.3	3.84 (3.69–4.00)
Women	8.0	4.1	37.1	7.08 (6.19–8.09)
JCCMS				
Men	12.0	10.3	50.2	4.16 (3.89–4.45)
Women	1.3	5.7	72.1	9.63 (8.17–11.36)
JCCMS (Proposed cut-off points for WC)				
Men	12.0	10.3	50.2	4.16 (3.89–4.45)
Women	3.4	5.0	53.0	7.40 (5.68–9.64)

*DM* diabetes mellitus, *IDF* International Diabetes Federation, *JIS* the Joint Interim Statement on metabolic syndrome definition, Asian cut-off points for *WC* waist circumference ≥90 cm in men or ≥80 cm in women, Proposed cut-off points for *WC* waist circumference ≥85 cm in men or ≥80 cm in women, *JCCMS* Japanese Committee of the Criteria for Metabolic Syndrome

^a^HRs are adjusted for age and tobacco use

### Sensitivity and specificity

Table 5 shows the sensitivity and specificity for prediction of incident DM in the next 5 years using MetS. In both sexes, the JIS criteria [8] had a higher sensitivity than the JCCMS criteria [10]. In men, a large increase in sensitivity (44.1 to 54.5 %), with only a moderate decrease of the positive predictive value (18.5 to 17.1 %), was seen in the JIS criteria when the WC cut-off point (90 cm) was substituted by our proposed value (85 cm). In women, the sensitivity increased (JCCMS, 13.3 to 25.8 %) when the Japanese obesity criteria (WC ≥90 cm) [10] was substituted by our proposed cut-off point (80 cm). The specificity for all criteria ranged from 80.6 to 98.9 %. The positive and negative predictive values were approximately ~28 and 95 %, respectively.

**Table 5:** Sensitivity and specificity of JIS and JCCMS criteria for the prediction of diabetes in the next 5 years

	JIS Asian	JIS New	JCCMS	JCCMS new
Sensitivity				
Men	44.1	54.5	37.7	37.7
Women	43.5	43.5	13.3	25.8
Specificity				
Men	85.7	80.6	90.0	90.0
Women	93.5	93.5	98.9	97.3
PPV				
Men	18.5	17.1	21.7	21.7
Women	17.9	17.9	28.2	23.8
NPV				
Men	95.4	96.0	95.2	95.2
Women	98.1	98.1	97.2	97.6

*JIS* the Joint Interim Statement on metabolic syndrome definition, Asian waist circumference ≥90 cm in men or ≥80 cm in women; New: our proposed waist circumference ≥85 cm in men or ≥80 cm in women; *JCCMS* Japanese Committee of the Criteria for Metabolic Syndrome, *PPV* positive predictive value, *NPV* negative predictive value

### Additional analyses

When having WC as an “optional” rather than “essential” criterion (modified JCCMS criteria), the sensitivity modestly increased from 37.7 to 41.7 % in men and from 13.3 to 14.2 % in women. The specificity slightly decreased from 90.0 to 89.7 % in men and from 98.9 to 98.8 % in women.

## Discussion

In the present prospective study of a large Japanese working population, we demonstrated that the optimal cut-off points of WC for predicting DM were 85 and 80 cm for men and women, respectively. In the comparison of the JCCMS criteria, the JIS criteria using the above WC cut-off points provided the highest sensitivity for predicting DM. To our knowledge, this is the first prospective study in Japan to assess the optimal WC cut-off points for predicting DM and to compare the predictive abilities of different MetS criteria for the development of DM.

In the present study, the incidence rate of DM was 14.7 per 1000 person-years for men and 6.5 per 1000 person-years for women. According to a systematic review and meta-analysis of Japanese studies [28], the incidence rates of DM showed varied considerably among studies (2.3–52.6 per 1000 person-years). This could be attributable to differences in background characteristics (e.g., age and gender), follow-up durations, and diagnostic procedures among studies. In line with the present findings, previous studies in the Japanese population also found a higher incidence of DM among men than among women [29, 30]. For instance, the incidence rate of DM was 20.9 per 1000 person-years for men and 9.8 per 1000 person-years for women in the Ibaraki Prefectural Health Study (age range: 40–79 years) [29].

Much controversy exist over the current Japanese cut-offs for WC (85 cm for men and 90 cm for women) [31], which have been adopted in the JCCMS. The results of the present ROC analysis indicate that the optimal cut-off points of WC for predicting the development of DM are 85 cm for men and 80 cm for women. Several studies in the Asian population have reported that the optimal WC cut-offs for predicting DM were 80–85 cm for men and 75–80 cm for women, based on cross-sectional data relating WC to the presence of DM [32–34]. A prospective cohort study in Korea also suggested that WCs of 85 cm for men and 80 cm for women were appropriate cut-offs to predict the development of DM [15]. Taken together, WC cut-off points of 85 cm for men and 80 cm for women may be suitable for the prediction of development of DM. Besides, a community-based cohort study in Japan suggested that the optimal WC cutoff points for predicting CVD were 90 cm for men and 80 cm for women [14]. Further prospective studies are required to confirm whether optimal cut-off points of WC for predicting DM are different from those for predicting CVD.

To determine whether, and to what extent, our proposed WC cut-off points (85 cm for men and 80 cm for women) can improve the performance of the MetS criteria for predicting DM, we further analyzed the impact of different WC cut-off points on the sensitivity and specificity of MetS for predicting the development of DM. In women, the sensitivity of the JCCMS criteria substantially increased from 13.3 to 25.8 % after substituting the original WC cut-off (90 cm) with our proposed cut-off (80 cm). Similarly, in men, the sensitivity of the JIS criteria increased from 44.1 to 54.5 % when the WC cut-off (90 cm) was substituted by our proposed value (85 cm). These findings suggest that a MetS criteria using WC cut-offs of 85 cm for men and 80 cm for women can identify a greater number of people with increased risks of DM than the cut-offs for the Asian population (for men) or the current Japan central obesity criteria (for women).

Several prospective studies outside Japan have investigated the predictability of MetS (defined by IDF, NCEP, WHO, and the European Group for the Study of Insulin Resistance) for the development of DM [4]. The Hong Kong Cardiovascular Risk Factor Prevalence Study showed that the sensitivity of the modified NCEP-ATP III (similar to the JIS criteria) tended to be higher compared with the IDF criteria (41.7 % versus 31.9 %) in the total population [3]. Further, another study in Beijing, China, also showed that the sensitivity of the modified NCEP-ATP III was higher than that of the IDF criteria (52 % versus 44 %) [35]. In our study, the JIS criteria (Asian WC cut-off values, 44.1 % for men and 43.5 % for women; proposed cut-off values, 54.5 % for men and 43.5 % for women) was more sensitive for predicting incident DM than the JCCMS criteria (Japanese central obesity criteria, 37.7 % for men and 13.3 % for women; proposed cut-off values, 37.7 % for men and 25.8 % for women), whereas both two criteria had similar positive predictive values (approximately ~28 %) and negative predictive values (>95 %). Therefore, the JIS criteria seems to have superior sensitivity to predict incident DM in East Asian populations.

Our additional analyses showed only a slight increase in sensitivity in both sexes when WC was treated as an optional component of the JCCMS criteria. This finding suggests that including WC as an “optional” component in the current JCCMS criteria has minimal impact on its predictability for DM. One possible explanation is that WC itself may not be a good risk predictor of DM. In fact, FPG has much higher ability to identify individuals with high risk of DM than do other components of the MetS [3, 36, 37].

### Limitations

The main strengths of our study include its prospective design, large sample size, and sufficient number of DM events. However, several limitations need to be considered. First, because the majority of study participants were employees of large companies, caution should be exercised in generalizing the present finding to workers in smaller-sized companies or non-working population. Second, the sample size for women was relatively small and the estimates obtained were unstable. Third, the methods of blood glucose and HbA1c measurements differed among the companies. Given the highest level of quality control achieved in all of the participating companies, however, measurement bias is unlikely. Measurement error is also inevitable in the assessment of exposures including WC and blood pressure. Such error usually occurs in random manner and thus might have attenuated the association. Fourth, because data on family history of DM and lifestyle other than smoking (e.g., diet and physical activity) were not collected in a standardized manner across the participating companies, we were unable to control for the effects of these confounders. Lastly, our data are not able to discriminate between type 1 and type 2 DM. However, given that the prevalence of type 1 DM is as low as 1.75 per 10,000 in Japanese adults aged 20 years and over [38], most cases in the present study maybe type 2 DM.

## Conclusion

In conclusion, the results of the present study show that WC cut-offs of 85 cm for men and 80 cm for women are appropriate for predicting incident DM in the Japanese population. Use of the JIS criteria detected more cases of incident DM compared to the JCCMS criteria.

## Availability of data and materials

Not applicable.

## Additional files

Additional file 1: Table S1. Baseline characteristics of participants with no history of diabetes stratified by metabolic syndrome and sex. (DOCX 16 kb) Additional file 2: Figure S1-2. Kaplan-Meier estimates of diabetes-free survival by metabolic syndrome and sex.pdf. Figure S1.Kaplan-Meier estimates of diabetes-free survival by metabolic syndrome (JIS criteria) and sex; Figure S2. Kaplan-Meier estimates of diabetes-free survival by metabolic syndrome (JCCMS criteria) and sex. (PDF 98 kb)

## Abbreviations

AUC: area under the curve
BMI: body mass index
DM: diabetes mellitus
HbA1c: glycated hemoglobin
HDL-C: high density lipoprotein-cholesterol
HR: hazard ratio
IDF: International Diabetes Federation
JCCMS: Japanese Committee of the Criteria for Metabolic Syndrome
J-ECOH: Japan Epidemiology Collaboration on Occupational Health
JIS: Joint Interim Statement
LDL-C: low-density lipoprotein-cholesterol
MetS: metabolic syndrome
NCEP-ATP III: National Cholesterol Education Program Adult Treatment Panel III
ROC: receiver operating characteristic
TCH: total cholesterol
TG: triglyceride
WC: waist circumference
