# Supplementary material for: Optimal waist circumference cut-off points and ability of different metabolic syndrome criteria for predicting diabetes in Japanese men and women: Japan Epidemiology Collaboration on Occupational Health Study
Source: BMC Public Health. 2016 Mar 3;16:220. doi: 10.1186/s12889-016-2856-9 (PMC4778284; doi:10.1186/s12889-016-2856-9)
Supplement: Additional file 1: — Table S1. Baseline characteristics of participants with no history of diabetes stratified by metabolic syndrome and sex. (DOCX 16 kb) [file 12889_2016_2856_MOESM1_ESM.docx]

|  | Metabolic syndrome, JIS criteria | |  | Metabolic syndrome, JCCMS criteria | |
| --- | --- | --- | --- | --- | --- |
|  | Without | With |  | Without | With |
| **Men** |  |  |  |  |  |
| N | 39,240 | 7,741 |  | 41,348 | 5,633 |
| Age (years) | 45.1±9.0 | 48.4±7.8* |  | 45.2±9.0 | 48.8±7.7* |
| BMI (kg/m2) | 23.0±2.7 | 26.6±3.2* |  | 23.1±2.8 | 26.7±3.0* |
| WC (cm) | 81.5±7.3 | 92.0±7.6* |  | 82.0±7.7 | 92.6±6.5* |
| FPG (mg/dl) | 95.7±8.4 | 104.2±8.4* |  | 96.1±8.3 | 104.3±10.3* |
| TCH (mg/dl) | 199.3±31.9 | 211.0±34.6* |  | 200.0±32.2 | 212.3±34.4* |
| TG (mg/dl) | 111.5±72.2 | 209.9±134.1* |  | 116.4±79.4 | 210.6±134.7* |
| LDL-C (mg/dl) | 119.3±29.1 | 127.5±31.1* |  | 119.6±29.3 | 128.1±30.7* |
| HDL-C (mg/dl) | 58.5±14.2 | 48.4±12.2* |  | 57.9±14.4 | 49.5±11.7* |
| SBP (mmHg) | 119.7±14.1 | 132.7±14.5* |  | 120.1±14.2 | 135.3±13.5* |
| DBP (mmHg) | 75.8±9.9 | 85.1±9.7* |  | 76.1±10.0 | 86.7±9.1* |
| Hypertension (%) | 14.8 | 51.0* |  | 15.5 | 59.2* |
| Smoking (%) | 40.6 | 41.8 |  | 40.9 | 40.0 |
| **Women** |  |  |  |  |  |
| N | 7,362 | 637 |  | 7,894 | 105 |
| Age (years) | 43.6±8.8 | 49.5±8.2* |  | 44.0±8.9 | 49.2±7.9* |
| BMI (kg/m^2^) | 21.2±3.0 | 26.6±3.2* |  | 21.5±3.3 | 30.2±4.3* |
| WC (cm) | 74.6±8.4 | 89.3±7.9* |  | 75.4±8.8 | 97.4±6.9* |
| FPG (mg/dl) | 90.3±7.5 | 101.1±9.1* |  | 91.0±8.0 | 103.9±10.3* |
| TCH (mg/dl) | 200.0±32.9 | 216.9±37.7* |  | 201.0±33.6 | 215.1±32.5* |
| TG (mg/dl) | 70.5±33.1 | 141.7±92.3* |  | 75.1±43.4 | 155.0±104.5* |
| LDL-C (mg/dl) | 112.3±29.2 | 134.1±32.7* |  | 113.8±30.0 | 134.1±29.6* |
| HDL-C (mg/dl) | 70.6±15.0 | 56.0±15.7* |  | 69.7±15.5 | 55.5±13.9* |
| SBP (mmHg) | 114.1±15.2 | 133.4±15.7* |  | 115.3±16.0 | 136.8±12.0* |
| DBP (mmHg) | 70.8±10.2 | 82.9±9.8* |  | 71.6±10.6 | 85.4±8.4* |
| Hypertension (%) | 7.9 | 43.5* |  | 10.1 | 61.9* |
| Smoking (%) | 10.9 | 10.1 |  | 10.8 | 13.3 |

Table S1 Baseline characteristics of participants with no history of diabetes stratified by metabolic syndrome and sex

Data was expressed as mean±SD or as percentages.

*Difference between groups is statistically significant (P < 0.05).

BMI: body mass index, WC: waist circumference, FPG: fasting plasma glucose, TCH: Total cholesterol, TG: triglyceride, LDL-C: low-density lipoprotein cholesterol, HDL-C: high-density lipoprotein cholesterol, SBP: systolic blood pressure, DBP: diastolic blood pressure, JIS: the Joint Interim Statement on metabolic syndrome definition, JCCMS: Japanese Committee of the Criteria for Metabolic Syndrome.
