# Supplementary material for: Optimal waist circumference cut-off points and ability of different metabolic syndrome criteria for predicting diabetes in Japanese men and women: Japan Epidemiology Collaboration on Occupational Health Study
Source: BMC Public Health. 2016 Mar 3;16:220. doi: 10.1186/s12889-016-2856-9 (PMC4778284; doi:10.1186/s12889-016-2856-9)

Figure S1 Kaplan-Meier estimates of diabetes-free survival by metabolic syndrome (JIS criteria) and sex

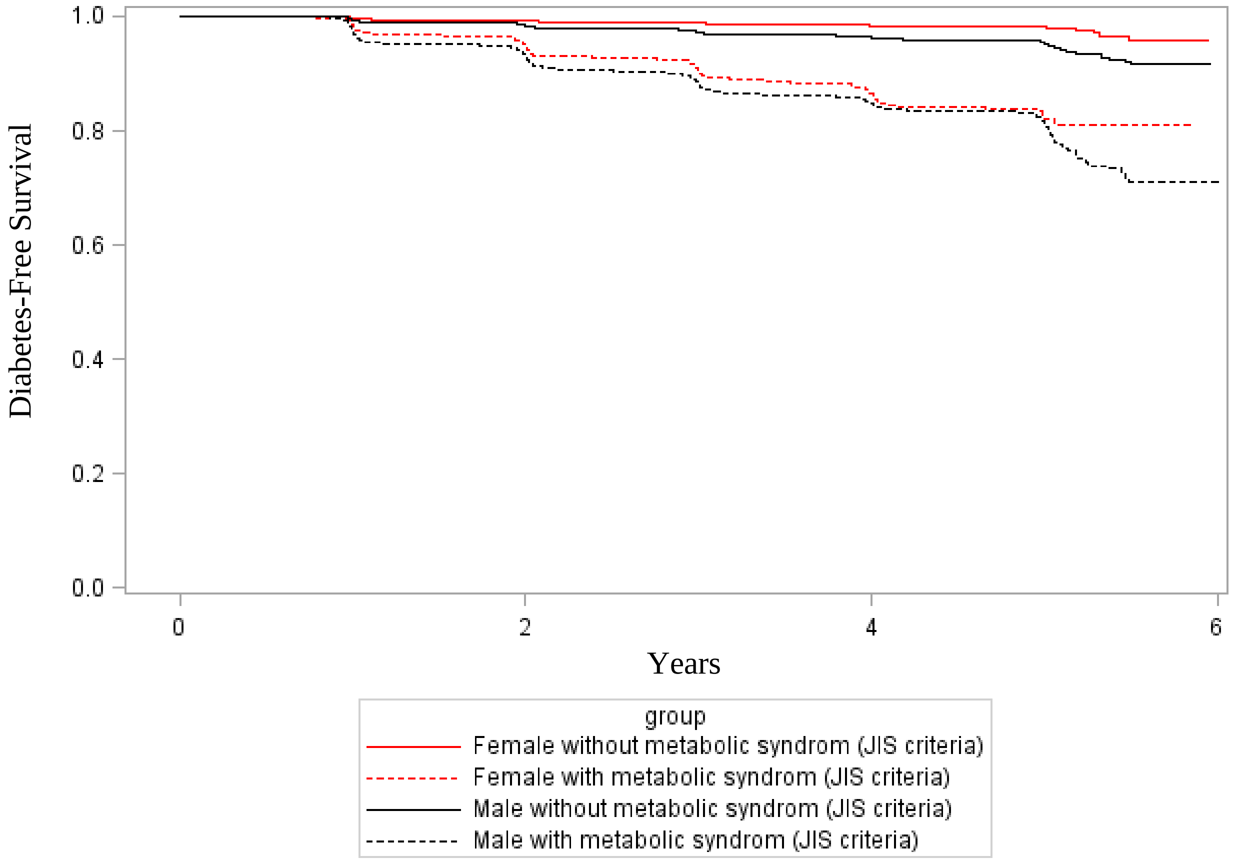

Figure S2 Kaplan-Meier estimates of diabetes-free survival by metabolic syndrome (JCCMS criteria) and sex

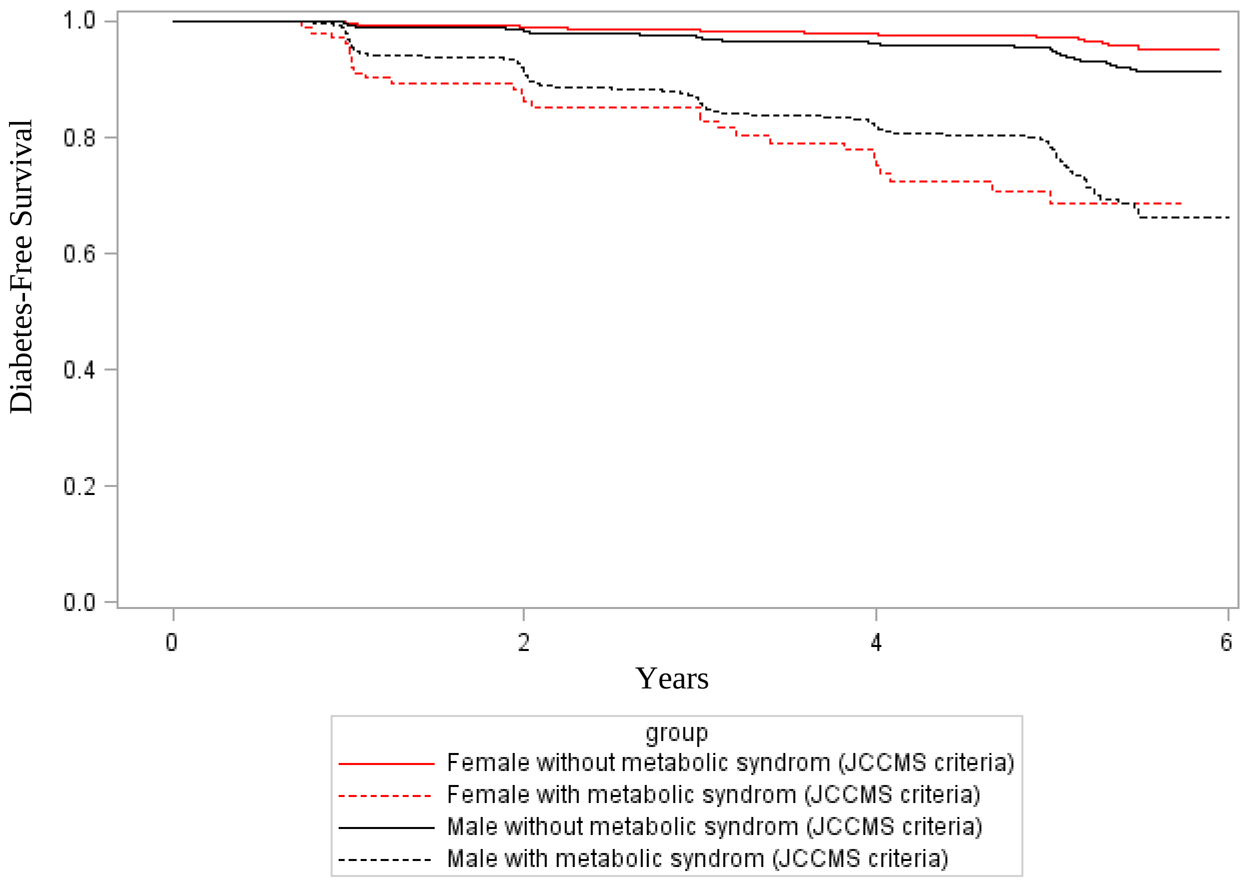

Supplement: Additional file 2: — Figure S1-2. Kaplan-Meier estimates of diabetes-free survival by metabolic syndrome and sex.pdf. Figure S1.Kaplan-Meier estimates of diabetes-free survival by metabolic syndrome (JIS criteria) and sex; Figure S2. Kaplan-Meier estimates of diabetes-free survival by metabolic syndrome (JCCMS criteria) and sex. (PDF 98 kb) [file 12889_2016_2856_MOESM2_ESM.pdf]
